# A dual dynamically cross-linked hydrogel promotes rheumatoid arthritis repair through ROS initiative regulation and microenvironment modulation-independent triptolide release

**DOI:** 10.1016/j.mtbio.2024.101042

**Published:** 2024-04-03

**Authors:** Tianyang Wang, Cheng Huang, Ziyuan Fang, Abudureheman Bahatibieke, Danping Fan, Xing Wang, Hongyan Zhao, Yajie Xie, Kun Qiao, Cheng Xiao, Yudong Zheng

**Affiliations:** a School of Material Science & Engineering, University of Science and Technology Beijing, Beijing, 100083, China; b Department of Orthopaedics, China-Japan Friendship Hospital, Beijing, 100029, China; c Beijing Key Laboratory of Research of Chinese Medicine on Prevention and Treatment for Major Diseases, Experimental Research Center, China Academy of Chinese Medical Sciences, Beijing, China; d China-Japan Friendship Clinical Medical College, Beijing University of Chinese Medicine, Beijing, China; e Institute of Clinical Medicine, China-Japan Friendship Hospital, Beijing, 100029, China; f Department of Emergency, China-Japan Friendship Hospital, Beijing, 100029, China

**Keywords:** Triptolide, ROS responsiveness, Macrophage polarization, Rheumatoid arthritis

## Abstract

High oxidative stress and inflammatory cell infiltration are major causes of the persistent bone erosion and difficult tissue regeneration in rheumatoid arthritis (RA). Triptolide (TPL) has become a highly anticipated anti-rheumatic drug due to its excellent immunomodulatory and anti-inflammatory effects. However, the sudden drug accumulation caused by the binding of “stimulus-response” and “drug release” in a general smart delivery system is difficult to meet the shortcoming of extreme toxicity and the demand for long-term administration of TPL. Herein, we developed a dual dynamically cross-linked hydrogel (SPT@TPL), which demonstrated sensitive RA microenvironment regulation and microenvironment modulation-independent TPL release for 30 days. The abundant borate ester/tea polyphenol units in SPT@TPL possessed the capability to respond and regulate high reactive oxygen species (ROS) levels on-demand. Meanwhile, based on its dense dual crosslinked structure as well as the spontaneous healing behavior of numerous intermolecular hydrogen bonds formed after the breakage of borate ester, TPL could remain stable and slowly release under high ROS environments of RA, which dramatically reduced the risk of TPL exerting toxicity while maximized its long-term efficacy. Through the dual effects of ROS regulation and TPL sustained-release, SPT@TPL alleviated oxidative stress and reprogrammed macrophages into M2 phenotype, showing marked inhibition of inflammation and optimal regeneration of articular cartilage in RA rat model. In conclusion, this hydrogel platform with both microenvironment initiative regulation and TPL long-term sustained release provides a potential scheme for rheumatoid arthritis.

## Highlights

• Borate ester-tea polyphenol units in SPT@TPL hydrogel exhibits sensitive ROS response and regulation behavior.
• Triptolide can be safely released from SPT@TPL hydrogel with microenvironment regulation-independent kinetics.
• ROS regulation and TPL release significantly alleviated macrophage inflammation in rheumatoid arthritis.

## Graphical abstract

**Figure:**
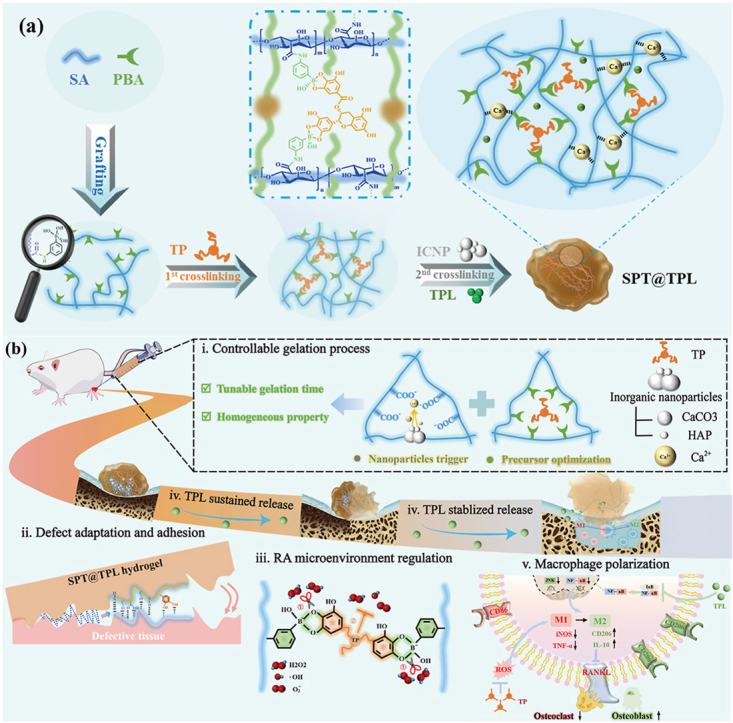

## 1 Introduction

Rheumatoid arthritis (RA) is a highly intricate autoimmune disease that typically presents with severe pain and swelling in the joints of the hands and feet. Long-term degenerative changes in bone cartilage can lead to bone erosion and osteoporosis, which can easily result in fractures and other progressive disabilities [1]. In addition, RA can also affect different parts of the body, including the heart, kidneys, respiratory system, and nervous system, causing a variety of non-specific extra-articular symptoms. This chronic disease, which affects 0.5 %–2.0 % of the world's population [2], has placed a heavy burden on patients' quality of life and psychological state.

The pathogenic pathways of rheumatoid arthritis (RA) are multifaceted and intricate, mainly centered on oxidative stress and inflammatory joint damage [3,4]. The accumulation reactive oxygen species (ROS) at the injured site leads to oxidative stress. On the one hand, it instigates detrimental effects on intracellular macromolecules like lipids, proteins and DNA [5]. On the other hand, it can also serve as a signaling molecule that induce inflammation and upregulate the HIF-1α/VEGF and Notch angiogenesis-related pathways, which are significant contributors to synovial hyperplasia and bone destruction [6]. Macrophages abnormally activation is pivotal in creating an inflammatory microenvironment and obstructing tissue regeneration. Macrophages are chemoattracted to the lesion site by damage signals, where they are polarized into pro-inflammatory phenotype by T cells and immune complexes [7], and produce a large number of pro-inflammatory factors such as TNF-α, IL-1β, IL-6, MMP. These factors bind to Toll-like receptors on the surface of macrophages and activate NF-κB, JNK, MAPK and other signals pathway [[8], [9], [10]], leading to the secretion of more inflammatory factors and continued infiltration of macrophages [11]. In addition, upregulated pro-inflammatory factors prevent the maturation of osteoblasts [12,13] and induce high expression of RANKL on the macrophages, which will bind to RANK and activate osteoclasts to erode bone [14,15], resulting in gradual defect expansion and hindered regeneration. Thus, regulating the high ROS microenvironment and inhibiting macrophages inflammation is a crucial target for RA treatment [16,17].

Currently, non-steroidal anti-inflammatory drugs [18], corticosteroids [19] and disease-modifying anti-rheumatic drug (DMARD) [20] are mainly used in clinic to alleviate acute symptoms and delay the degeneration of osteochondral and joint deformity. Nonetheless, these drugs are unable to bring out a complete cure and may potentially yield unexpected side effects [21]. Triptolide (TPL) is an epoxy diterpene lactone compound extracted from Tripterygium wilfordii, a traditional Chinese medicine. It exhibits superior anti-inflammatory properties and immune regulation compare to the conventional *anti*-rheumatoid drug, methotrexate [22]. Firstly, TPL significantly lessens the synthesis of macrophage inflammatory protein (MIP), monocyte chemoattractant protein (MCP-1) and other chemokines [23], preventing macrophages from migrating to the diseased area. Secondly, TPL can inhibit the activation of intracellular transcription factor NF-κB, thereby reducing the secretion of pro-inflammatory factors and angiogenesis-related factors, which is beneficial to restoring the balance between osteogenesis and osteoclasts in the lesion and reducing bone erosion [24]. However, TPL's extreme liver, kidney and reproductive toxicity, even at low concentrations, and poor water solubility severely limits its clinical application [25,26]. Therefore, a safe, stable and lasting drug delivery solution for TPL requires further investigation.

In recent years, many efforts have been made to develop sustained-release delivery routes, including microneedles [27], liposomes [28], nanoparticles [29] and hydrogels [30]. Yang et al. [31] showed that mannose-modified polymer vesicles can be targeted and absorbed at RA lesion sites. Methotrexate was then slowly released to induce macrophage repolarization to M2 phenotype, thereby alleviating inflammation and preventing synovium damage. Deng et al. [32] developed an MMP-responsive RGD-PEG nanoparticle to deliver tripterine, which can reduce RA inflammation and restore bone function balance by selectively inducing apoptosis of macrophages and osteoclasts. Zhao et al. [33] fused the M1 macrophage membrane to the surface of M2 macrophage exosomes, and black phosphorus nanosheets were introduced to prepare hybrid nano-vesicles targeting inflammatory factors. The nano-vesicles eliminated inflammatory cells under near-infrared irradiation to reduce RA inflammatory response. All these new drug delivery methods focused on encapsulation and targeting. Among them, hydrogel has particularly promising features such as irregular defect adaptation, in-situ delivery and the extensive extracellular matrix-like high-water content are advantageous for the regeneration of articular cartilage [34,35]. Nonetheless, the increased concentration of ROS in the RA microenvironment not only results in cellular damage, but also impairs the structure of hydrogel owing to its strong oxidative mechanisms [36,37], reducing its retention time at the injury site, and needs to be lowered and removed in a timely manner. The incorporation of a ROS response and scavenging systems in hydrogels enables the regulation of ROS levels in the microenvironment on demand [38], however, this sensitive response behavior often results in a loose internal structure and sudden drug release [39], which will instantly increase the local concentration of TPL, thereby enhancing the risk of toxicity. Hence, investigating the method that can sensitively regulate the high ROS microenvironment without interfering the stable release of TPL could be a promising approach for RA treating. As far as we know, this strategy appears to have received minimal attention in previous research.

**Fig. 1:**
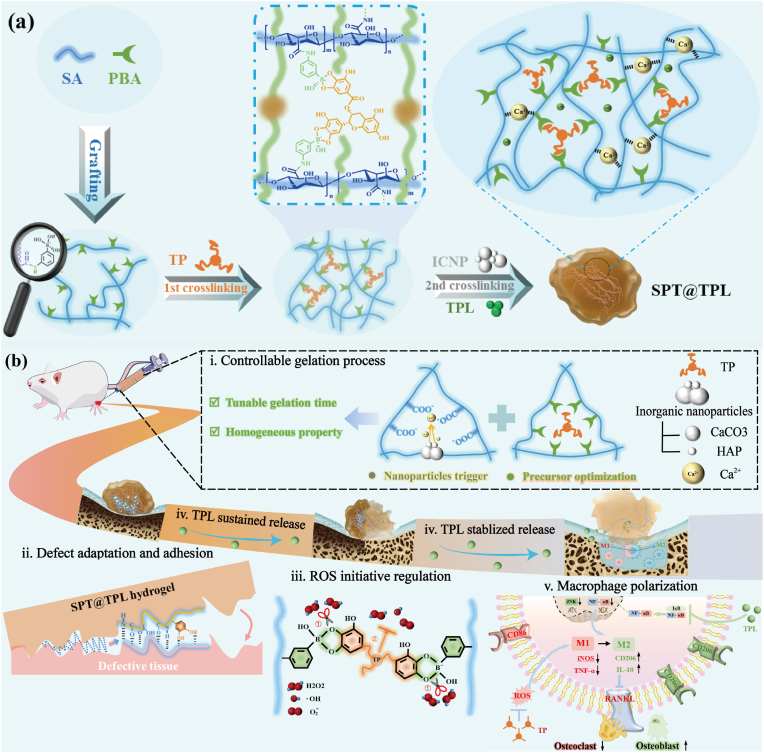
Synthesis and function schematic of SPT@TPL hydrogel for rheumatoid arthritis treatment.

In this paper, we developed a dual dynamically cross-linked sodium alginate hydrogel (SPT@TPL) for ROS regulation and in-situ TPL delivery in RA microenvironment (Fig. 1). Based on the “borate ester precursor optimization” and “inorganic nanoparticle trigger” synthesis strategy, SPT@TPL exhibited adjustable gelation times, robust tissue adhesion and self-healing properties. Moreover, SPT@TPL can actively respond to and scavenge excess ROS in the RA microenvironment, and since the hydrogel could immediately formed numerous intermolecular hydrogen bonds for structure repair after borate ester fractured, the ROS regulation process would not trigger the sudden release of TPL, which greatly reducing the risk of it exerting toxicity. The synergistic effect of on-demand ROS regulation and sustained-release of TPL effectively down-regulated oxidative stress, repolarized the macrophages to anti-inflammatory phenotype and accelerated the regeneration of articular cartilage in the RA rat.

## 2 Materials and methods

### 2.1 Materials

Sodium alginate (SA, M/G = 1:2, 90 %), triptolide (TPL, 98 %), 4-(4,6-dimethoxytriazin-2-yl)-4-methylmorpholine hydrochloride (DMTMM, 97 %) and 3-Aminophenylboronic acid (PBA, 98 %) were purchased from Macklin (Shanghai, China). Tea polyphenols (TP, 99 %), potassium iodide (KI, 99.5 %), 30 % hydrogen peroxide (H_2_O_2_), safranin O (95 %), and ferrous sulfate (FeSO_4_, 90 %) were purchased from China National Pharmaceutical Group Reagent Co., Ltd. Nano-calcium carbonate (CaCO_3_, 99.9 %, 50 nm), nano-hydroxyapatite (HA, 99 %, 20 nm) and DPPH free radical scavenging kit were obtained from Aladdin (Shanghai, China). Gluconolactone (GDL, 99 %) was purchased from Beijing Tongguang Chemical Co., Ltd.

### 2.2 Synthesis and characterization of SA-PBA precursor

1.0 g SA (5 mmol) was dissolved in deionized water (50 mL), then 0.7 g, 1.4 g and 2.8 g DMTMM were added, the pH of the solution was adjusted to 6.5 by 1 M HCl and stirred for 30 min. PBA with the same molar number as DMTMM was subsequently added to the solution and reacted at room temperature for 24 h. After that, the mixture was dialyzed with deionized water for 3 days (MWCO = 8000–12,000 Da), and then freeze-dried to obtain SA-PBA. The product was labelled as SA-PBA0.5, SA-PBA1 and SA-PBA2 respectively according to the dosage ratio of SA to DMTMM.

The viscosity of SA-PBA was determined by Hakke falling ball viscometer (002–7580, HAAKE, Germany). Before the test, the temperature was controlled at 25 °C via a liquid circulator, and then the falling time was measured with a standard ball made of nickel-iron alloy. The dynamic viscosity was calculated by the following equation:

(1) $$\begin{array}{c} \eta \;\left(\text{mPa}∙s\right)=K\left(\rho_{1}-\rho_{2}\right)\times t \end{array}$$

In the equation, K stands for the spherical constant of the standard sphere, while t represents the falling time of the sphere, ρ_1_ and ρ_2_ denotes the density of the standard sphere and the SA-PBA at the measurement temperature.

### 2.3 Preparation of SPT and SPT@TPL hydrogel

Typically, the freeze-dried SA-PBA was dissolved in deionized water, after that, tea polyphenols and the inorganic composite nanoparticles (compose of nano calcium carbonate and nano hydroxyapatite in equal amounts) were dispersed in the solution by sonication. Finally, 2 wt% GDL was added for secondary crosslinking to form SPT hydrogel. For the synthesis of SPT@TPL, the triptolide was dissolved in ethanol and initially added to SA-PBA, following by the same steps as in SPT synthesis, the final concentration of TPL in the hydrogel was 10 μg/mL. Additionally, the single cross-linked SPT hydrogel was also prepared by simply mixing SA-PBA and TP solution according to the same concentration in SPT and SPT@TPL.

The components of SPT and SPT@TPL hydrogels are shown in Table S1.

### 2.4 Tunable gelation process of SPT hydrogel

The gelation process of SPT with varying tea polyphenol and inorganic composite nanoparticle concentrations was analyzed by tube inversion method. Every 30 s, invert the Eppendorf tube containing 5 mL sample, judge the progress of the sol-gel transition process through the system flow state and record the initial gelation time and final gelation time.

### 2.5 Chemical structure and morphology of SA-PBA and SPT

The chemical structure of SA-PBA and SPT hydrogel was examined by Fourier transform infrared spectroscopy (TENSOR II, Bruker, Germany). The scanning range was set to 4000-600 cm^−1^ with a resolution was 4 cm^−1^. Moreover, SA-PBA with different substitution degrees was dissolved in deuterium oxide (D_2_O), and ^1^H NMR spectra were detected through nuclear magnetic resonance hydrogen spectroscopy (AVANCE 400 MHz, Bruker, Germany). The substitution degree of PBA was calculated by the integral ratio of the characteristic peaks of PBA (between 7.5 and 8.0 ppm) and sodium alginate (4.0 ppm).

SA-PBA and SPT were frozen at −80 °C and freeze-dried for 24 h. The resultant freeze-dried samples were fixed using conductive glue and then coated with gold. A field emission scanning electron microscope (S-4800, Hitachi, Japan) was utilized to observe the microstructure of the samples at an accelerating voltage of 10 kV.

### 2.6 Physicochemical properties of SPT hydrogel

#### 2.6.1 Rheology

The rheological properties of SPT were tested by a rheometer (MCR92, Anton Paar). Cut the gel into discs with a diameter of 25 mm and a thickness of 2 mm, and the storage modulus and loss modulus of SPT was obtained through frequency scanning ranging from 0.1 to 15 Hz. The self-healing capability was evaluated by alternating strain scanning at a fixed angular frequency of 10 rad/s. Each 30 s period of alternating low (1 %) to high (500 %) strain oscillations was repeated four times.

#### 2.6.2 Mechanical properties

The compression properties of SPT’ and SPT were measured by texture analyzer (TA-XT plus, Stable Micro systems, UK). Each sample were cut into rectangles of 2 cm × 1 cm, and loaded at a speed of 4 mm/s at room temperature with P75 compression plate to obtain the compressive stress-strain curve and compressive modulus.

#### 2.6.3 Adhesion properties

The adhesive properties of hydrogel were evaluated through a shear lap test. Similar to the method reported in the past [40], SPT was placed between two pieces of 1 cm × 3 cm freshly porcine skin with a contact area of 1 cm × 1 cm. Porcine skin was stretch at a speed of 0.5 cm/min using the TA-XT plus texture analyzer (Stable Microsystems, Godalming, UK) until the hydrogel broke. The adhesive strength was calculated by the subsequent equation:

(2) $$\begin{array}{c} Adhesive\;strength\;\left(Pa\right)=\frac{F_{\max}}{A} \end{array}$$

where F_max_ is the maximum force during the stretching process, and A is the contact area between the gel and the porcine skin. Furthermore, integrating the force over the tensile displacement and normalizing the contact area in order to obtain the adhesion energy:

(3) $$\begin{array}{c} Adhesive\;energy\;\left(J/m^{2}\right)=\frac{\int Fdx}{A} \end{array}$$

#### 2.6.4 Swelling and degradation

Incubate SPT hydrogel in PBS simulated body fluid at 37 °C for a period of time to reach swelling balance. The initial mass and the mass after soaking of the sample was denoted as m_0_ and m_t,_ respectively. The swelling rate of the hydrogel was calculated as:

(4) $$\begin{array}{c} swelling\;ratio\;\left(\%\right)=\frac{m_{t}-m_{0}}{m_{t}}\times 100\% \end{array}$$

SPT with the same specifications were immersed in PBS for 30 days. The initial mass of the sample weighed by an electronic precision balance was W_0_, and the mass after t time was W_t_. The remaining weight of the hydrogel can be calculated by the following equation：

(5) $$\begin{array}{c} remaining\;weight\;\left(\%\right)=\frac{W_{0}-W_{t}}{W_{t}}\times 100\% \end{array}$$

### 2.7 Free radical scavenging ability

The antioxidant capacity of SPT@TPL hydrogel was characterized by ROS and RNS scavenging experiments, The specific operations were as follows:

#### 2.7.1 H_2_O_2_ scavenging

KI assay was employed to determine the scavenging effect of hydrogels on hydrogen peroxide, modified from previous work [41].0.5 g SA, SPT1@TPL and SPT2@TPL hydrogels were added to 5 mL H_2_O_2_ (0.2 mM). The blank group used the same amount of deionized water instead of the hydrogel, and for the control group, the same amount of deionized water was used to substitute the H_2_O_2_. Three groups were then incubated in the dark at 37 °C for 30 min. Finally, 2 mL KI (60 mM) and 2 mL HCl (20 mM) were added into the supernatant and left for 5 min. The absorbance of each solution was measured at 351.5 nm by UV spectrophotometer, and the scavenging rate of hydrogen peroxide could be calculated according to the following equation:

(6) $$\begin{array}{c} S_{H2O2}\;\left(\%\right)=\frac{A_{Hydrogel}-A_{Blank}}{A_{Control}-A_{Blank}}\times 100\% \end{array}$$

#### 2.7.2 Hydroxyl radical scavenging

Fenton assay was employed to determine the scavenging effect of hydrogels on hydroxyl radical, modified from previous work [42]. 1.85 g SA, SPT1@TPL and SPT2@TPL hydrogels were added to a mixture of 0.83 mL Safranin O (0.36 mg/mL), 1 mL FeSO_4_ (2 mM) and 1.33 mL H_2_O_2_ solution (6 %), and supplemented with deionized water to 5 mL. After incubating in a water bath (55 °C) for 30 min and cooled, the absorbance of three groups at 553.2 nm were measured. By applying the following equation, the scavenging effect of hydroxyl radical can be obtained:

(7) $$\begin{array}{c} S_{∙OH}\;\left(\%\right)=\frac{A_{Hydrogel}-A_{Blank}}{A_{Control}-A_{Blank}}\times 100\% \end{array}$$

#### 2.7.3 DPPH scavenging

The DPPH scavenging kit was used to test the ability of hydrogels for DPPH radicals scavenging. SA, SPT1@TPL and SPT2@TPL hydrogels (50 μL) were incubated with 950 μL DPPH ethanol solution in the dark. The absorbance was measured at 515 nm via UV spectrophotometer, and the scavenging effect of DPPH radicals can be calculated according eto the following equation:

(8) $$\begin{array}{c} S_{DPPH}\;\left(\%\right)=\frac{A_{Hydrogel}-A_{Blank}}{A_{Control}-A_{Blank}}\times 100\% \end{array}$$

### 2.8 TPL release and kinetic mechanism in different microenvironment

Firstly, ethanol solutions with concentrations of 0.5 μg/mL, 1 μg/mL, 5 μg/mL, 10 μg/mL, 20 μg/mL and 50 μg/mL TPL were prepared. The absorbance at 220.4 nm was measured by a UV spectrophotometer, and the standard curve of TPL was then plotted.

The release curve of SPT@TPL in normal and high ROS simulated body fluids were both investigated. The hydrogel samples were incubated in 37 °C PBS buffer with or without 0.1 mM H_2_O_2_, and the supernatant was taken for UV spectroscopy measurement on the 5th, 10th, 15th, 20th, 25th and 30th days, and the concentration of TPL at each time point could be calculated based on the standard curve.

In order to clarify the kinetic mechanism of TPL release in different microenvironments, Zero-order, First-order, Higuchi and Rigter-Peppas models were used to fit the release curve.

### 2.9 Cell-count-Kit8 (CCK-8) and live cell staining

1.0 g SPT and SPT@TPL were incubated in 10 mL of complete DMEM/FBS medium at 37 °C for 48 h to extract the hydrogel. Bone marrow mesenchymal stem cells (BMSCs) were seeded in a 48-well plate with a density of 1 × 10^5^/well and cultured for 12 h. Then the culture medium was replaced by the hydrogel extract. 10 μL of CCK-8 reagent were added to the wells at day-1, 3 and 7, then incubated in the dark for 4 h. The absorbance at 450 nm was measured by a microplate reader to determine the cytotoxicity the hydrogels. Similarly, after the CCK-8 experiment at each time point, live cells were stained by calcein and observed under an inverted fluorescence microscope (BX-50, Olympus).

### 2.10 In-vitro antioxidant capacity

BMSCs (1 × 10^5^ cells per well) were seeded onto the surface of SPT@TPL hydrogels and incubated in a 48-well plate with 1 mL DMEM/FBS medium for 12 h. Then, the medium was replaced with 1 mL DMEM/FBS medium that contained 0.1 mM H_2_O_2_ and continued to culture for 24 h. CCK-8 and live/dead cell staining were used as above to characterize the growth status of BMSCs in a high ROS environment.

Intracellular reactive oxygen species levels were assessed through DCFH-DA staining. BMSCs were cultured on the surface of SPT@TPL and co-incubated with 1 mL DMEM/FBS culture medium in a 48-well plate. After 12 h, the culture medium was substituted with 1 mL DMEM/FBS medium containing 0, 0.1, or 1 mM H_2_O_2_, and the incubation continued for 1 h and 4 h. The culture medium in the well was disposed at each time point and replaced with 200 μL DMEM medium including 10 μM DCFH-DA. It was then incubated in the dark for 30 min. The distribution and quantity of ROS in the cells were observed by the confocal microscope (Zeiss).

### 2.11 In-vitro anti-inflammation capacity

RAW264.7 cells were seeded in a 48-well plate at a density of 5 × 10^4^/well. Lipopolysaccharides (LPS, 1 μg/mL) was added to the wells and incubated for 4 h to induce an inflammatory phenotype. After that, they were co-cultured with SPT and SPT@TPL while the same volume of DMEM medium was used in the control group instead. After incubation for 7 days, immunofluorescence staining was performed on the M1 cell's marker iNOS and the M2 cell's marker CD206, and the relative intensity of each fluorescence was calculated by ImageJ.

Furthermore, RAW264.7 cells from each group were collected, and the real-time fluorescence quantitative polymerase chain reaction (RT-qPCR) was used to detect the relative expression of iNOS, TNF-α, Arg-1, and IL-10 genes in the cells.

### 2.12 Animal experiments

All animal experiments were approved by China-Japan Friendship Hospital (zryhyy-21-11-02). The RA model was induced by type II collagen. Briefly, fifteen Sprague-Dawley rats were injected subcutaneously with 200 μg of bovine type II collagen emulsified in complete Freund's adjuvant on day 0. After Seven days, 100 μg of bovine type II collagen emulsified in incomplete Freund's adjuvant was injected subcutaneously again to enhance immunity. Fifteen SD rats with swollen ankle and toe were divided into control group, SPT group and SPT@TPL group regardless of gender. The rats were received anesthesia via intraperitoneal injection of pentobarbital (35 mg/kg). All of their legs, tails and backs were depilated and disinfected by iodophor. A model of rheumatoid arthritis cartilage injury was constructed by cutting through the skin and subcutaneous layer on both left legs, followed by drilling a 1.0 mm deep, 2.0 mm diameter circular hole within the trochlear groove of the medial femoral condyle. The control group was sutured directly without any other treatment, while 0.25 mL hydrogel was injected into the damaged area in SPT group and SPT@TPL group before the wound was sutured.

#### 2.12.1 Medical imaging characterization

The rats were sacrificed after 2 months, and the articular cartilage was subsequently extracted for Micro-CT scanning and 3D reconstruction. An area was randomly selected at the defect site, and the software of Micro-CT was then used to analysis the bone-related parameters, including bone mineral density (BMD), bone volume fraction (BV/TV) and the number of trabeculae (Tb. N.). In addition, magnetic resonance imaging (MRI) was also applied to observe and assess the damage and the regeneration of articular cartilage.

#### 2.12.2 ICRS and histological staining

Rats were anesthetized and sacrificed two months after modeling and the articular cartilage of each group was collected for ICRS scoring. Articular cartilage in the modeling area was fixed with 4 % paraformaldehyde for two days, rinsed with PBS and decalcified in 10 % EDTA for 4 weeks. After graded dehydrated with ethanol, the samples were embedded in paraffin and sectioned at 5 μm thickness. Histological staining was performed with hematoxylin-eosin, Masson, and safranin-fast green, and observe under a light microscope.

### 2.13 Statistical analysis

All quantitative data were described as mean ± standard deviation. Univariate analysis of variance was analyzed via IBM SPSS 27 software to perform the Tukey test. *P < 0.05, **p < 0.01 or ***p < 0.001 were considered as statistically significant, while ns meant no significant difference.

## 3 Results and discussion

### 3.1 Synthesis and characterization of SA-PBA precursor

**Fig. 2:**
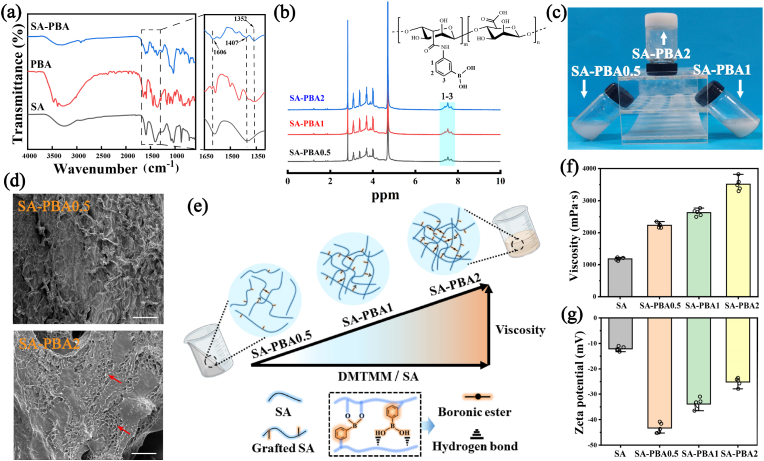
Characterization of structure and properties of SA-PBA precursor. (a) FT-IR spectrum of SA-PBA. (b) ^1^H NMR of SA-PBA with different grafting rates. (c) SA-PBA precursor solution state. (d) Morphology of SA-PBA0.5 and SA-PBA2 (scale bar: 100 μm). (e) Schematic diagram of viscosity mechanism of SA-PBA. (f) Viscosity of SA-PBA. (g) Zeta potential of SA-PBA.

Upon activation by DMTMM, the carboxyl group of SA was amidated with the amino group of PBA, and a viscous and opaque SA-PBA was obtained. The chemical structure of the product was analyzed through FT-IR spectrum (Fig. 2a). It can be seen that the O–H stretching vibration peak of SA has been altered from 3000 to 3600 cm^−1^ to a broad peak of 2000–3600 cm^−1^, which was probably due to the grafting of PBA, greatly increasing the hydrogen bonds in the system. The characteristic peaks of PBA were all reflected in the spectrum, in which the absorption peak at 1606 cm^−1^ was caused by benzene ring's skeleton vibration [43], while the tensile vibration peak of B–O at 1352 cm^−1^ [44] and the tensile vibration of C–N at 1407 cm^−1^ represented the formation of the amide bond [45,46]. All the results demonstrate the successful grafting of PBA.

^1^H NMR was applied to characterize the chemical structure of SA-PBA with different grafting ratios, which also proved that PBA was grafted onto the sodium alginate (Fig. 2**b**). The identifiable peak of sodium alginate was found within 3.2–4.0 ppm region [47], and the proton signal between 7 and 8 ppm was considered to be the peak of benzene ring in PBA [48]. By comparing the area of the two integral peaks, it can be determined that the degrees of substitution of phenylboric acid in SA-PBA0.5, SA-PBA1 and SA-PBA2 were 11.6 %, 38.3 % and 45.7 %, respectively.

The viscosity of the precursor solution is a crucial factor in the hydrogel molding process. Mixing with high-viscosity precursor solution may lead to a rapid and uneven gelation, which can seriously damage the uniformity of the hydrogel network and affect its physiochemical as well as drug release properties. As shown in Fig. 2**f**, the viscosity of SA-PBA was significantly higher than that of SA, reaching 2240 mPa s, 2915 mPa s and 3103 mPa s respectively. It is important to note that a quantity of boronic ester and intermolecular hydrogen bonds can also be formed between SA-PBAs, even without the extra crosslinking agent, resulting in a substantiated increase in their viscosity (Fig. 2**e**). Zeta potential (Fig. 2**g**) confirmed the existence of this interaction. As the grafting ratio increases, the zeta potential of SA-PBA decreased gradually, which indicates that the electrostatic repulsion energy between SA-PBA particles decreased and the interaction enhanced. Fig. 2**c** shows the solution state of SA-PBA, it can be observed that the SA-PBA0.5 and SA-PBA1 had a satisfactory fluidity, however, with an increased grafting rate, the viscosity of the solution gradually raised, SA-PBA2, in particular, exhibited a high viscosity to the extent that the solution adopted a gel-like state, making it almost immobile even when inverted. From the SEM, it can be seen that there was already a little network in the microstructure of SA-PBA2 with a higher grafting rate (Fig. 2**d**), which proved detrimental to the hydrogel molding process. Based on the above results, SA-PBA0.5 and SA-PBA1 were selected for the subsequent research.

### 3.2 Tunable gelation process and morphology of SPT hydrogel

**Fig. 3:**
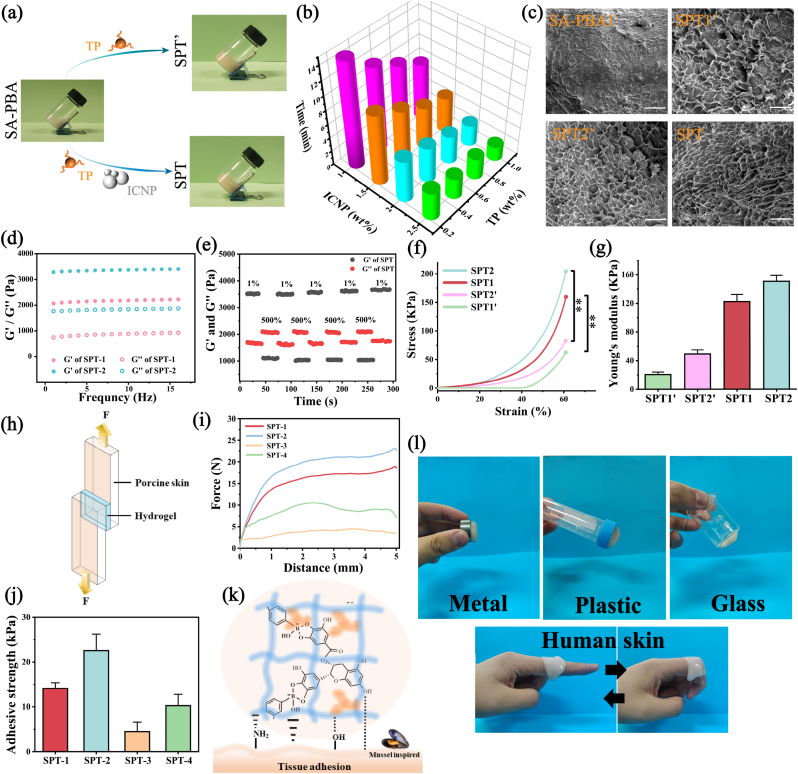
Characterization of SPT hydrogel. (a) SPT gelation process. (b) Statistics of gelation time. (c) Morphology of SA-PBA, single-crosslinked SPT’ and double-crosslinked SPT (Scale bar: 100 μm). (d) Frequency-modulus curve. (e) Recovery behavior of storage modulus (G′) and loss modulus (G″) of SPT under alternating strain. (f) Compressive stress-strain curves of SPT’ and SPT. (n = 3, **P < 0.01) (g) Compressive modulus of SPT’ and SPT. (h) Schematic diagram of shear lap experiment. (i) Adhesion mechanism of SPT. (j) Adhesion-displacement curve between SPT and porcine skin. (k) Adhesion strength between SPT and porcine skin. (l) SPT can closely adhere to metal, plastic, glass and human skin.

Based on the SA-PBA precursor, the SPT hydrogel (Fig. 3**a**) was prepared through borate ester and ionic crosslinking. Theoretically, the incorporation of tea polyphenols into SA-PBA leads to the rapid formation of dynamic borate ester bonds between the catechol and pyrogallol groups. Previous studies had shown that this covalent bond can be formed in less than 30 s at room temperature [42,49]. Nevertheless, this speedy gelation method has negative implications, including the uncontrollable process and unstable gel properties, which is similar to the calcium ion cross-linking sodium alginate [50,51]. To ensure the controllability of the dual crosslinking process, a combined strategy of “precursor concentration control” and “nanoparticle trigger” was adopted in this study. On the one hand, low concentration of tea polyphenols (<1 wt%) was applied to prevent uneven gelation on the solution surface, and on the other hand, inorganic composite nanoparticles (ICNP) composed of nano-calcium carbonate and nano-hydroxyapatite were introduced as the tunable ionic cross-linking trigger. By adjusting the concentration of tea polyphenols from 0.2 wt% to 1.0 wt% as well as the mass fraction of composite nanoparticles from 1 wt% to 2.5 wt%, the gelation time of SPT can be flexibly controlled between 2 min and 14 min (Fig. 3**b**), which is of great importance for obtaining a homogeneous gel and adapting to the different gelation processes required for minimally invasive injection surgery.

Fig. 3**c** shows that the single crosslinked SPT’ had a randomly distributed and interconnected network compared to the un-crosslinked SA-PBA, this can be attributed to the formation of borate bonds between tea polyphenols and phenylboric acid. Moreover, the introduction of the second crosslinking in SPT hydrogel yielded a dense network composed of electrostatic interaction and borate bond, leading to a higher crosslinking density and smaller pore size, which is beneficial for the gradual release of triptolide encapsulated in the hydrogel.

### 3.3 Rheology and mechanical properties

The rheological properties of the SPT hydrogel were analyzed via frequency scanning and amplitude scanning using a rheometer. It can be seen that the storage modulus (G′) of SPT-1 and SPT-2 was maintained at 2.05 kPa and 3.23 kPa within the frequency range of 0.1–15 Hz (Fig. 3**d**), and both of them were higher than loss modulus (G″), indicating excellent elasticity under the test frequency. The self-healing property of SPT was characterized by cyclic strain scanning test (Fig. 3**e**). When the shear strain of 500 % was exerted, the storage modulus of the gel dropped below the loss modulus and the network was destroyed. Nevertheless, upon transitioning to low strain (1 %), the storage modulus and loss modulus returned to their original levels, and after four cycles, the self-healing efficiency remained nearly 90 %. The dual dynamically crosslinked network enabled the hydrogel to quickly recombine with the surrounding groups and healed into a new network after being destroyed by external stimuli. Upon injection into the cartilage defect, this hydrogel can withstand and recover the structural damage caused by frequent joint activities, which will prolong the hydrogel retention in the defect and extend the release time of triptolide, enhancing the potential for effective and durable treatment of RA.

Articular cartilage in physiological environment has the function of continuously bearing loads and absorbing stress. Therefore, hydrogel injected into a cartilage defect should possess adequate mechanical properties to withstand consistent compressive loads [52]. The SPT hydrogel is cross-linked by borate ester and electrostatic interaction, and both mechanisms positively affect the mechanical properties of composite hydrogel. Fig. 3**f, g** depicts the compressive behavior of single cross-linked hydrogels (SPT-1′ and SPT-2′) and double cross-linked hydrogels (SPT-1 and SPT-2). The result demonstrates that the increased TP content led to higher crosslinking density and improved compression performance both in SPT’ and SPT, and all hydrogels can resistance up to 60% strain without being destroyed. Compared with SPT-1′ and SPT-2′, the compression modulus of SPT-1 and SPT-2 reached 122.2 KPa and 150.7 KPa, respectively. This considerable improvement was not only due to the introduction of the second cross-linking, but also to the presence of inorganic composite nanoparticles which did not react fully in SPT after the sol-gel transformation. These nanoparticles compensate for the micropores and defects of the network, provide additional ionic cross-linking and effectively disperse external loads, leading to a significant improvement in the mechanical properties of the hydrogel [53].

### 3.4 Adhesion properties

After injecting hydrogel into the cartilage defect of rheumatoid arthritis, frequent joint activity may increase the risk of detachment [54]. Therefore, good tissue adhesion is necessary for hydrogel to remain in the defect and stand a long-term therapeutic effect. The adhesion property of SPT hydrogel was assessed by a shear lap test on porcine skin (Fig. 3**h**). According to the force-displacement curve, the adhesion strengths (Fig. 3**i**, **j**) of SPT-1, SPT-2, SPT-3 and SPT-4 were calculated to be 13.56, 20.01, 8.14 and 11.49 Kpa, respectively. The adhesion strength and adhesion energy (Fig. S1) both enhanced as the dosage of coupling agent and tea polyphenols content increased. This is due to the higher density of the mussel-inspired interaction within the hydrogel (Fig. 3**k**), and the reversible dynamic structure based on PBA-Tea polyphenol allowed for a vast number of hydrogen bonds, metal coordination and cation-π non-covalent forces between tissue interface [55]. Fig. 3**l** demonstrates that the SPT hydrogel could closely adhere to metal, glass, plastic and human skin, showing excellent interfacial adhesion, which will help to form a close interface between hydrogel and tissue defect to allow triptolide to provide long-term therapeutic benefits for rheumatoid lesions.

### 3.5 In-vitro degradation and TPL release

**Fig. 4:**
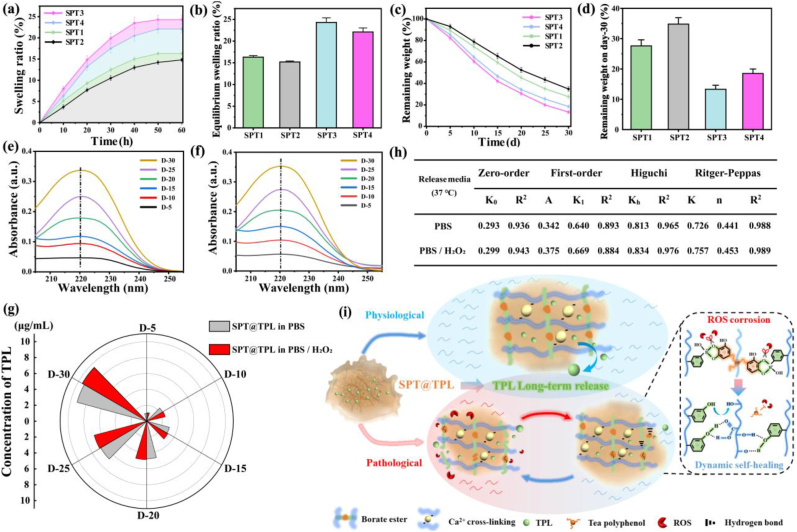
Degradation and drug release of SPT@TPL hydrogel. (a) Swelling curve and (b) Equilibrium swelling ratio of SPT. (c) Degradation curve and (d) Retained mass at day-30 of SPT. (e) TPL release curve in simulated body fluid. (f) TPL release curve in pathological simulated body fluid (adding 0.1 mM H_2_O_2_ to simulate high ROS microenvironment in RA). (g) The release amount of TPL from SPT@TPL in different microenvironments. (h) Fitting results of TPL release from SPT@TPL in different microenvironments. (i) Schematic diagram of TPL release mechanism in different microenvironments.

SPT@TPL hydrogel undergoes swelling followed by gradual degradation when exposed to a bodily fluid environment. Swelling experiments revealed that the equilibrium swelling rates of SPT-1, SPT-2, SPT-3 and SPT-4 are 16.3 %, 15.2 %, 23.7 % and 22.1 %, respectively (Fig. 4**a, b**). All the hydrogels exhibited comparably low swelling rates, which negated the possibility of excessive squeezing of the hydrogel into surrounding tissues post-implantation. At the same time, it was observed that the augmenting of PBA grafting rate and TP content lowered the swelling efficiency of the hydrogel. This could be attributed to the amplified density of borate ester dynamic network in SPT, which in turn reduced the water retention capacity and led to a decrease in the equilibrium swelling rate.

The degradation curve of SPT hydrogel (Fig. 4**c, d**) in simulated body fluid demonstrated that after a month, the mass retained by SPT-1, SPT-2, SPT-3 and SPT-4 stand for 27.6 %, 34.8 %, 13.3 % and 18.5 %, respectively. The degradation process of SPT composite hydrogel was more stable than that of sodium alginate hydrogel formed by ionic crosslinking. This is due to the stronger binding energy of the double crosslinking network itself, which in turn prevented monovalent ions in the solution from replacing calcium ions complexed by sodium alginate. Taking into account the homogeneity and degradation of the hydrogel, the drug release was studied by SPT-1 hydrogel encapsulated with triptolide.

Triptolide (TPL) is insoluble in water, so it was dissolved in ethanol to determine the standard curve. Fig. S2 shows that the fitting curve of TPL concentration and absorbance had a high level of accuracy, which can be used as the standard curve for subsequent experiments. As shown in Fig. 4**e, g**, the drug release behavior of SPT@TPL hydrogel in normal simulated body fluid was tested. On day-5, the absorbance of TPL was below 0.1 with a TPL concentration of 0.98 μg/mL once conversion. On the day-10, the concentration increased to 2.33 μg/mL, and on day-15, it rose further to 2.99 μg/mL. The minimal difference indicated that the concentration of TPL in the previous 15 days was insignificant, providing proof of SPT@TPL's effective drug loading and encapsulation. Over the next 15 days, the TPL release rate remained basically stable, which may be beneficial for the tissue to gradually adapt to the drug and better exert therapeutic effect. On the day-30, the release concentration of TPL in the solution accumulated to 9.17 μg/mL. These results suggest that SPT@TPL hydrogel can slowly and continuously release drugs, thus holding promise for long-term treatment of rheumatoid arthritis.

To further investigate the TPL release with high ROS levels, PBS containing 0.1 mM hydrogen peroxide was used instead of the normal simulated body fluid [56]. As shown in Fig. 4**f, g**, the quantity and speed of TPL release were marginally higher than that in normal simulated body fluids, this is because hydrogen peroxide could break the connection between phenylboronic acid and polyphenols in SPT@TPL hydrogel, thereby relaxing the second cross-linking network and hastening the release of TPL to some extent. However, it should be note that the ROS response process did not widen the gap of TPL release in two environments. This could be attributed to the fact that, once the borate ester was broken by ROS, the residual phenolic hydroxyl groups were able to establish numerous intermolecular hydrogen bonds with hydroxyl groups or carboxyl groups in SA, which facilitated the restructuring and healing of the dynamic structure in the hydrogel, thereby staving off the swift release of internal TPL. This suggest that SPT@TPL hydrogel can also achieve long-term TPL release in a high ROS microenvironment of rheumatoid arthritis, Moreover, the hydrogel's response to ROS did not negatively impact TPL release (Fig. 4**j**), which shows that this release process is microenvironment-regulation-independent.

In order to clarify the release mechanism of TPL in both normal and high ROS environment, four kinetic models including Zero-order, First-order, Higuchi and Rigter-Peppas were employed to fit the release curve. According to Fig. 4**h**, the release of TPL in normal simulated body fluids was closer to the Higuchi model (R^2^ = 0.965), while the characteristic parameter “n” of the release mechanism in the Rigter-Peppas model was less than 0.45, which shows that the release of TPL predominantly followed the Fick diffusion law, and that the dense double cross-linked network managed the rate of drug release. However, at a high ROS level, the value for “n” in the Rigter-Peppas model exceeded 0.45, indicating that the release kinetics in this environment was more inclined to non-Fick diffusion law, and the release of TPL was influenced by the drug diffusion and the hydrogel skeleton dissolution [57], which was consistent with the conclusion that TPL release was slightly elevated in high ROS environment.

### 3.6 Free radical scavenging ability

Under normal physiological conditions, reactive oxygen species (ROS) and reactive nitrogen species (RNS) may function as second messenger molecules, encouraging intracellular and intercellular signal communication [58], but in the microenvironment of rheumatoid arthritis, excessive ROS can directly lead to cell apoptosis, oxidation stress and inflammation. It is crucial to suppress or eliminate these in time [59]. To demonstrate the capacity of hydrogels in scavenge ROS and RNS, SPT1@TPL and SPT2@TPL hydrogels were selected for a series of antioxidant tests.

**Fig. 5:**
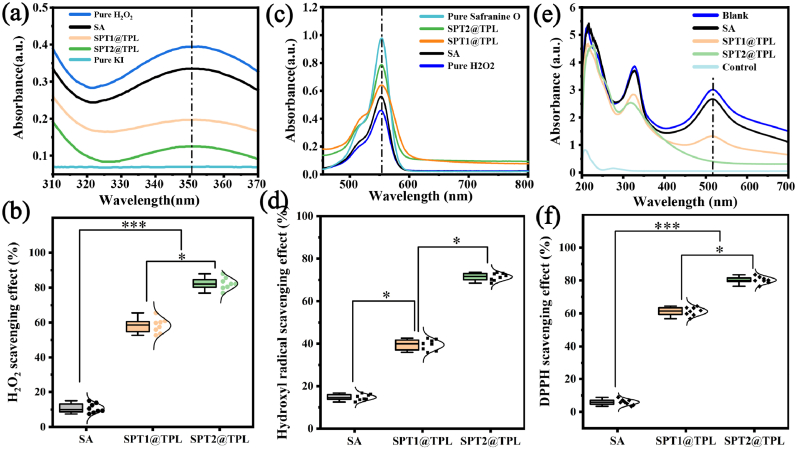
Free radical scavenging capacity of SPT hydrogel. (a) & (b) Hydrogen peroxide scavenging curve and scavenging rate. (c) & (d) Hydroxyl radical scavenging curve and scavenging rate. (e) & (f) DPPH radical scavenging curve and scavenging rate. (n = 8, *P < 0.05, **P < 0.01 and ***P < 0.001).

Hydrogen peroxide, an active oxygen species commonly found in the lesion microenvironment, can react with KI to generate I^3−^, which has a potent absorption at 351.5 nm, and when the hydrogen peroxide is removed, the absorption intensity of I^3−^ will obviously decrease. As shown in Fig. 5**a, b**, the absorbance of SPT1@TPL and SPT2@TPL at 351.5 nm was significantly lower than that of SA as well as the control group. Their scavenging efficiency for hydrogen peroxide was calculated to reach 60.8 % and 82.7 % respectively.

The ability of the hydrogel to scavenge hydroxyl radicals was assessed by the Fenton reaction. The hydroxyl radicals created by the Fenton reaction can discolor safranine O and reduce its absorption intensity at 553.2 nm. Fig. 5**c, d** shows that the absorbance of SPT1@TPL and SPT2@TPL was close to that of the blank group, indicating that the hydrogel effectively shielded safranine O from hydroxyl radicals within 30 min and their hydroxyl radical scavenging rates can reach 37.2 % and 70.9 %.

DPPH is a typical nitrogen radical, and the single electron held by its nitrogen atom can be captured by antioxidants, which reduces its maximum absorbance at 517.5 nm. As shown in Fig. 5**e, f**, SPT1@TPL and SPT2@TPL considerably decreased the absorption of DPPH at 517.5 nm, and their scavenging efficiencies were calculated to be 63.5 % and 79.9 %, respectively, indicating their positive effects on scavenging DPPH free radicals.

To sum up, SPT@TPL hydrogel exhibited a satisfactory antioxidant capacity far stronger than that of sodium alginate itself. Therefore, SPT@TPL hydrogel can respond sensitively and remove excessive free radicals after being implanted into rheumatoid articular cartilage defects, hence reducing oxidative stress and offering a favorable microenvironment for tissue regeneration, and also provide an appropriate condition for the long-term retention in the defects.

### 3.7 Biocompatibility of SPT@TPL

**Fig. 6:**
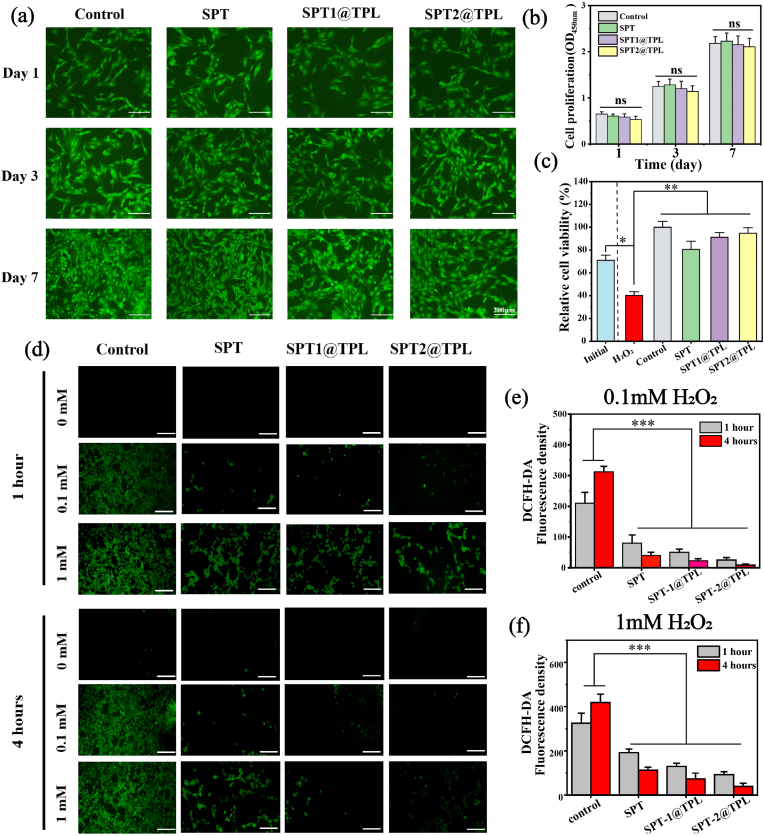
Cytocompatibility and antioxidant properties of SPT@TPL hydrogel. (a) Fluorescence micrographs of BMSCs co-cultured with SPT@TPL on the 1st, 3rd and 5th days (Scale bar: 200 μm). (b) Cell proliferation measured by CCK-8. (n = 3, scale bar: 100 μm) (c) Relative cell viability of BMSC incubated with 0.1 mM H_2_O_2_ on the surface of SPT@TPL for a day. (n = 3) (d) DCFH-DA staining after BMSCs incubated with different concentrations of H_2_O_2_ for 1 and 4 h (scale bar: 100 μm) (e) & (f) Fluorescence intensity of DCFH-DA calculated by Image J (n = 5). (*P < 0.05, **P < 0.01, ***P < 0.001, ns represent no significant difference).

CCK-8 and live cell staining were applied to evaluate the cytotoxicity of hydrogels on the 1st, 3rd and 7th day. Fig. 6**a, b** demonstrates satisfactory spreading and growth of BMSCs in all groups. Additionally, the OD value of SPT and SPT@TPL hydrogels on the 1st and 3rd day exceeded 90 % of the control group, suggesting that the double cross-linked network of the hydrogel can effectively seal TPL for a period of time without evident toxicity.

### 3.8 In-vitro antioxidant properties

To establish the protective efficacy of SPT@TPL hydrogel on cells under oxidative stress conditions, hydrogen peroxide was employed as ROS source and co-cultured with BMSCs and hydrogel. Based on the results of CCK-8 and Live/Dead staining (Fig. 6**c,** S3), it was obviously that the cells treated with H_2_O_2_ were in an inferior growth state, with a large-scale cell damage and death. However, compared to the day before hydrogen peroxide treatment, the quantity of cells in both SPT1@TPL and SPT2@TPL group had increased, and their proliferation rates were close to those in the control group, which were 28.5 % and 16.6 % respectively. Therefore, it can be inferred that SPT@TPL had eliminated the adverse impacts of H_2_O_2_ on cells through its antioxidant capabilities and promoted their vitality and reproduction under high ROS level. Furthermore, DCFH-DA staining was performed to clarify the mechanism that SPT@TPL hydrogel can protect BMSCs from damage in a high ROS environment. Fig. 6**d, e, f** show that the control group incubated with H_2_O_2_ displayed intense green fluorescence, indicating that a high level of ROS in BMSCs. However, after co-culturing with the SPT@TPL hydrogel, the fluorescence intensity of DCFH-DA decreased significantly. After 4 h, only a minuscule amount of green fluorescence was observed in the BMSCs, especially in SPT2@TPL group. This result suggests that SPT@TPL hydrogel effectively reduced the overproduction ROS in BMSCs by scavenging free radicals in the external environment on-demand, thereby preventing cell apoptosis during oxidative stress.

### 3.9 In-vitro anti-inflammatory ability

**Fig. 7:**
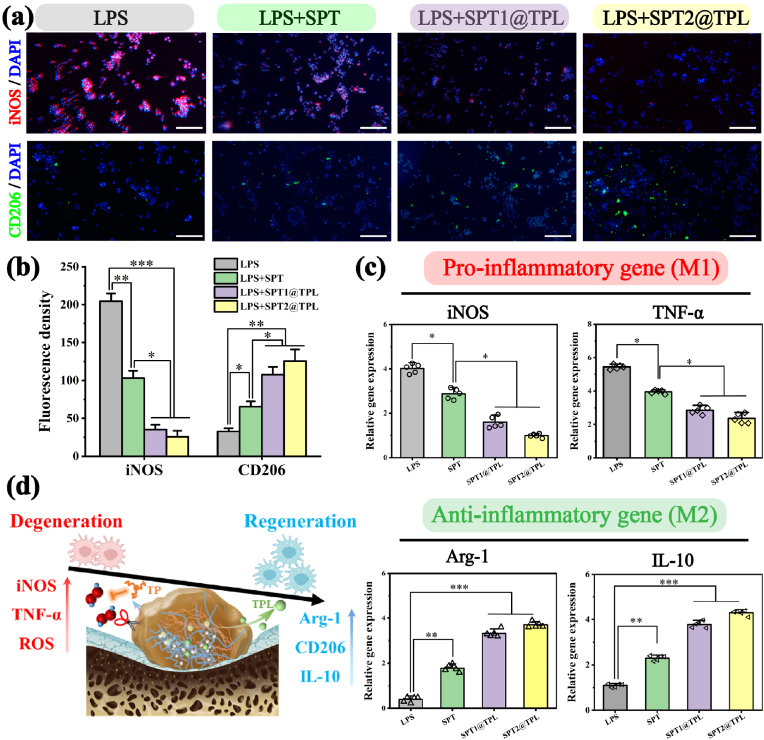
In-vitro anti-inflammatory of SPT@TPL hydrogel. (a) Immunofluorescence staining of macrophages co-cultured with SPT@TPL hydrogel and (b) Statistics of fluorescence intensity of two phenotypes. (n = 3, scale bar: 100 μm) (c) Relative expression of pro-inflammatory genes and anti-inflammatory genes compared to GADPH in macrophages co-cultured with SPT@TPL hydrogel. (n = 5) (d) Schematic diagram of the mechanism of phenotypic transformation of M1 macrophages induced by SPT@TPL. (*P < 0.05, **P < 0.01, ***P < 0.001).

Rheumatoid arthritis can invade articular cartilage. Abnormally activated M1 macrophages at the lesion site expel a large number of pro-inflammatory factors to induce the inflammation. An extend inflammatory response is the primary cause of irreversible damage to cartilage and bone loss surrounding joint mediated by bone immunity [60]. Regulation of macrophage transformation into an anti-inflammatory phenotype will accelerate the secretion of interleukin 10 (IL-10), transforming growth factor β (TGF-β) and bone morphogenetic protein (BMP), which will benefit the protection and regeneration of articular cartilage. To demonstrate the ability of SPT@TPL to trigger macrophages phenotypic transformation, LPS was applied firstly to polarize macrophages into M1 type in advance to simulate the inflammation of rheumatoid arthritis, and then they were co-cultured with SPT and SPT@TPL hydrogels. After 7 days, the immunofluorescence staining (Fig. 7**a, b**) revealed that macrophages treated with LPS expressed high levels of inducible nitric oxide synthase (iNOS), which was a clear indicator of cellular inflammation. Meanwhile, macrophages co-cultured with SPT hydrogel showed significantly reduced levels of iNOS, suggesting that the inflammation induced by macrophage was alleviated, possibly due to SPT's sensitively responsive and downregulatory effects on excess ROS released by M1 macrophages. However, in the SPT@TPL group, almost no red fluorescence can be observed, the intensity of which was only 17.2 % and 12.6 % of that in the LPS group. It should also be noted that the fluorescence intensity of these two groups was significantly lower than that of SPT group as well, and this difference, except for ROS response regulation, should be attributed more to the release of TPL. Through their common and safe synergy, the inflammation of macrophages was basically inhibited. CD206 staining demonstrated that both SPT and SPT@TPL hydrogels augmented the proportion of M2 macrophages compared to the LPS group, indicating that these hydrogels could also significantly induce the phenotypic transformation of macrophages. RT-qPCR was used to further quantify the relative contents of pro-inflammatory and anti-inflammatory genes in macrophages (Fig. 7**c**). Similar to the staining results, the relative expressions of pro-inflammatory genes iNOS and TNF-α in macrophages co-cultured with SPT1@TPL and SPT2@TPL were significantly down-regulated, while anti-inflammatory genes Arg-1 and IL-10 were up-regulated. All the results indicate that the SPT@TPL hydrogel effectively inhibits inflammation and reprograms macrophages into an anti-inflammatory phenotype, which in turn creates an environment that supports the regeneration of articular cartilage in the affected area of rheumatoid arthritis, and this ability may due to the sustained-release of triptolide and the ROS responsive release of tea polyphenols (Fig. 7**d**).

### 3.10 In-vivo RA model establishment and medical imaging evaluation

**Fig. 8:**
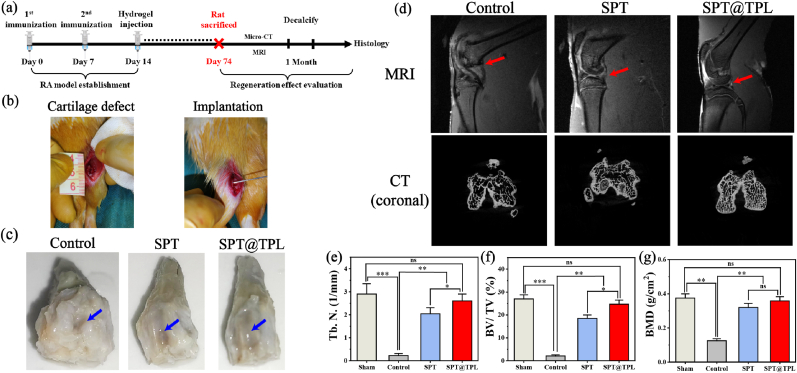
(a) Schematic diagram of animal experiment schedule. (b) A round cartilage defect module with a depth of 1.0 mm and a diameter of 2.0 mm above the trochlear groove (Left) and injection of SPT@TPL hydrogel (Right). (c) Gross observation of cartilage two months after modeling. (d) Micro-CT coronal images and MRI of femoral condyle of rats after two months. Two months after injury, the levels of bone-related parameters of rats in each group: (e) Trabecular Number, (f) Bone Mineral Density and (g) Bone Volume Fraction. (n = 5, *P < 0.05, **P < 0.01, ***P < 0.001, ns represent no significant difference).

As shown in Fig. 8**a, b**, a rat model of rheumatoid arthritis cartilage injury was established. The defect was created by drilling a circular hole with a depth of 1.0 mm and a diameter of 2.0 mm located in the trochlear groove of the femoral condyle, and hydrogel was injected immediately after the drilling. Fig. 8**c** shows the gross observation of the articular cartilage in each group after two months. The articular cartilage of the control group exhibited severe deformity, characterized by the evident degenerative lesions and fibrosis deformation, and it was impossible to observe the location of the trochlear groove. The morphology of articular cartilage in SPT and SPT@TPL groups displayed favorable results, as the modeling surface remained smooth, but some injury can still be observed in SPT group, while the modeling injury in SPT@TPL group had essentially recovered.

In order to assess the efficacy of hydrogel in repairing cartilage defects of rheumatoid arthritis, MRI and Micro-CT were applied to evaluate the changes in cartilage and subchondral bone after two months. The MRI (Fig. 8**d**) revealed significant synovial hyperplasia in the control group and a marked absence of the typical structure of a healthy knee joint. The joint cavity was filled with proliferative synovial tissue, whilst the inflammation caused by rheumatoid arthritis completely eroded and destroyed the epiphyseal line of the femur and tibia, and the femoral condyle defect did not heal at all. More seriously, it was difficult to observe normal cartilage in the non-modeling area as well, indicating that this destructive behavior may spread to the adjacent healthy tissues. In the SPT group, minor synovial hyperplasia was noticed at the joint capsule, accompanied by a certain degree of joint structure defects. The clarity of the epiphyseal line at the femoral tibia suggested a partial improvement in rheumatoid inflammation, and partial healing with uneven tissue thickness in the defect at the trochlear groove of the femoral condyle. Nevertheless, the presence of a cartilage surface discontinuity at the femoral tibia indicated that SPT hydrogel had limited effectiveness in treating rheumatoid arthritis. In the SPT@TPL group, there was a decrease in the articular cavity hyperplasia, and the shape was basically normal as well. The epiphyseal line at the end of the femur and tibia was relatively clear and complete, and the cartilage surface was complete and continuous. The defect on the femoral condyle healed well, and the new tissue formed at the defect was close to the surrounding normal cartilage tissue. These results show that SPT@TPL hydrogel can effectively inhibit the further development of rheumatoid arthritis and repair articular cartilage. The 3D reconstruction of CT image (Fig. S4) demonstrates that the sparsity and wide separation of trabeculae in the bone marrow cavity of the control group, which were notably less than those in the other two groups. Conversely, the trabeculae in the experimental group became increasingly dense, indicating that SPT and SPT@TPL hydrogels can efficiently impede the bone destruction caused by rheumatoid inflammation.

Based on to the 2D-CT images (Fig. 8**d,** S4), it can be judged that both SPT and SPT@TPL groups possessed superior bone tissue morphology in comparison to the control group, and SPT@TPL group had obtained particularly obvious bone regeneration. The osteochondral parameters of the three groups were quantitatively analyzed through Micro-CT data. It is important to note that the bone erosion of rheumatoid arthritis mainly affects trabecular bone, leading to long-term bone loss and osteoporosis [61]. The statistical results of trabecular bone number (Tb. N.) in each group showed that (Fig. 8**e**), the bone of RA rats had been severely damaged after two months as the trabecular bone number was only 10 % of that of healthy rats. Meanwhile, the Tb. N levels of rats treated with SPT and SPT@TPL hydrogel achieved 2.1 and 2.6, respectively, which equated to 82 % and 95 % of healthy rats, indicating that SPT hydrogel can safeguard trabecular bone against erosion by rheumatoid arthritis particularly when loaded with triptolide. Besides, bone volume fraction (BV/TV) and bone mineral density (BMD) were measured to analyze the regeneration of articular cartilage. Similar to the above results, the BV/TV and BMD levels in RA rats were only 2 % and 0.13 g/cm^2^ (Fig. 8**f, g**), indicating severely bone erosion and no regeneration, however, the SPT hydrogel had a positive therapeutic effect on bone injury, with its BV/TV and BMD can reach more than 80 % of normal rats. In the group administered with triptolide (SPT@TPL), the BMD level of rats had increased to 0.35 g/cm^2^, which was comparable to the healthy rats (0.37 g/cm^2^), indicating highly favorable therapeutic outcomes.

### 3.11 ICRS and in-vivo histological evaluation

**Fig. 9:**
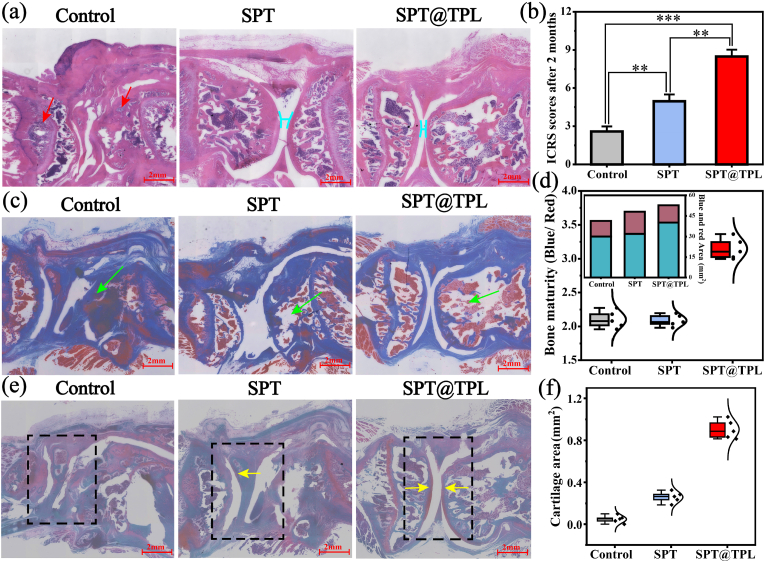
Articular cartilage score and histological staining. (a) H-E staining. (b) ICRS score. (c) Masson staining. (d) Statistics of bone mass and bone maturity in Masson staining. (e) Safranin-fast green staining. (f) Area of new cartilage on the cartilage surface. (For interpretation of the references to colour in this figure legend, the reader is referred to the Web version of this article.)

As depicted in Fig. 9**a**, H-E staining revealed significant pathological damage in the control group with limited healthy tissue observed. Moreover, a significant quantity of inflammatory cells was visible in the synovium (red arrows), indicating the destructive impact of rheumatoid arthritis on articular cartilage. However, both the SPT and SPT@TPL groups exhibited relatively intact tissue morphology, in the latter group, the surface of the new cartilage appeared more continuous and smoother, with a noticeable histological feature (blue line). This can be attributed to the synergistic effect of microenvironment regulation and triptolide, which leaded to a minimal infiltration of inflammatory cells in the synovial tissue. Consequently, the SPT@TPL hydrogel effectively alleviated the inflammatory response induced by rheumatoid arthritis. ICRS results (Fig. 9**b**) further demonstrate that the articular cartilage scores of the two hydrogel groups were higher than those of the control group. Notably, the SPT@TPL group attained the highest score and exhibited the most effective repair effect.

Rheumatoid arthritis causes ongoing damage to the bones. Masson staining was performed on the bone tissue, and the area as well as the ratio of blue region (mature type I collagen) and red region (newborn type I collagen) were counted to evaluate the bone mass and maturity. The bone marrow and new bone were difficult to observe, and the bone tissue layer was significantly thinner compared to the other two groups, as depicted in Fig. 9**c, d**, in the control group. The SPT group displayed higher bone density and a significant presence of new type I collagen, suggesting active bone regeneration. However, due to the immaturity of the new bones, the ratio of blue area to red area was relatively lower. It is noteworthy that the control group exhibited higher bone maturity than the SPT group, this anomaly may due to the lower overall bone mass with no type I collagen regeneration in the control group, resulting in a higher area ratio. In contrast, Masson staining in the SPT@TPL group revealed an even and dense accumulation of bone marrow in the bone marrow cavity, with minimal new type I collagen and predominantly mature type I collagen, exhibiting the greatest bone mass and maturity. These results demonstrate that both the SPT and SPT@TPL hydrogels can facilitate the repair of bone tissue injuries, of which the SPT@TPL hydrogel showed faster progress in bone maturity.

Safranin-fast green staining (Fig. 9**e**) was utilized to assess the damage and repair of cartilage tissue. It is evident that the control group had severe cartilage damage, making it difficult to observe normal cartilage tissue. In the SPT group, the cartilage layer where the model was built was visible, although the cartilage damage induced by modelling was only generally recovered, but no patches of cartilage tissue were formed. Additionally, various degrees of cartilage hyperplasia and inflammatory cells could still be observed in the non-modeled area. The SPT@TPL group displayed the lowest inflammation level in the cartilage tissue, with a generally healthy cartilage layer and few hyperplasia regions (green arrows). Most importantly, a rather thin cartilage layer grew in the modeled area. Furthermore, the area of new cartilage on the cartilage surface of each group was quantified (Fig. 9**f**). In line with the staining results, the SPT@TPL group exhibited a considerably wider expanse of new cartilage compared to the other two groups. These results conclusively illustrate the SPT@TPL hydrogel's capability to prevent the progression of rheumatoid arthritis, reduces cartilage destruction, and promotes tissue regeneration.

## 4 Conclusions

In this research, a double cross-linked hydrogel was developed for rheumatoid arthritis microenvironment regulation and TPL sustained-release. SPT@TPL could adapt and adhere tightly to articular cartilage defects without being easily dislodged, and achieved up to 30 days of TPL release. In vitro free radical scavenging and drug release experiments showed that SPT@TPL could respond to and effectively remove excessive reactive oxygen species in the RA microenvironment. Importantly, the robust self-healing capacity of the hydrogel ensured that the regulation of microenvironment would not negatively impact the TPL release process, which was confirmed through the drug release kinetics fitting test. Furthermore, the SPT@TPL hydrogel exhibits satisfactory biocompatibility, the tea polyphenols released in response to ROS stimulation and the sustained release of TPL jointly reprogram M1 macrophages into M2 phenotype at the lesion side, which inhibited the expression of inflammatory factors and promoted the regeneration of articular cartilage defects in RA. This regeneration effect was further proved via the medical imaging and histology in the rat model of rheumatoid arthritis. Therefore, this study provides a potential approach for in-situ and safe delivery of extremely toxic TPL as well as a promising option for rheumatoid arthritis treatment.

## CRediT authorship contribution statement

**Tianyang Wang:** Writing – review & editing, Writing – original draft, Methodology, Investigation, Data curation, Conceptualization. **Cheng Huang:** Visualization, Validation, Supervision, Methodology, Investigation. **Ziyuan Fang:** Writing – original draft, Supervision, Methodology, Investigation, Data curation, Conceptualization. **Abudureheman Bahatibieke:** Supervision, Methodology. **Danping Fan:** Methodology. **Xing Wang:** Methodology. **Hongyan Zhao:** Methodology. **Yajie Xie:** Data curation, Methodology, Visualization. **Kun Qiao:** Data curation, Methodology, Visualization. **Cheng Xiao:** Conceptualization, Funding acquisition, Investigation, Methodology, Resources, Supervision, Validation. **Yudong Zheng:** Visualization, Funding acquisition, Resources, Supervision, Validation.

## Declaration of competing interest

The authors declare that they have no known competing financial interests or personal relationships that could have appeared to influence the work reported in this paper.

## Data Availability

Data will be made available on request.
