# Supplementary material for: A dual dynamically cross-linked hydrogel promotes rheumatoid arthritis repair through ROS initiative regulation and microenvironment modulation-independent triptolide release
Source: Mater Today Bio. 2024 Apr 3;26:101042. doi: 10.1016/j.mtbio.2024.101042 (PMC11040138; doi:10.1016/j.mtbio.2024.101042)
Supplement: Multimedia component 1 [file mmc1.docx]

Supporting Information

**A dual dynamically cross-linked hydrogel promotes rheumatoid arthritis repair through ROS initiative regulation and microenvironment modulation-independent triptolide release**

Tianyang Wang ^a,1^, Cheng Huang ^b,1^, Ziyuan Fang ^a,1^, Abudureheman Bahatibieke ^a^, Danping Fan ^c^, Xing Wang ^d^, Hongyan Zhao ^c^, Yajie Xie ^a^, Kun Qiao ^a^, Cheng Xiao ^e, *^ Yudong Zheng ^a, **^

^a^ School of Material Science & Engineering, University of Science and Technology Beijing, Beijing 10083, China

^b^ Department of Orthopaedics, China-Japan Friendship Hospital, Beijing 100029, China

^c^ Beijing Key Laboratory of Research of Chinese Medicine on Prevention and Treatment for Major Diseases, Experimental Research Center, China Academy of Chinese Medical Sciences, Beijing, China

^d^ China-Japan Friendship Clinical Medical College, Beijing University of Chinese Medicine, Beijing, China

^e^ Institute of Clinical Medicine, China-Japan Friendship Hospital, Beijing 100029, China; Department of Emergency, China-Japan Friendship Hospital, Beijing 100029, China.

^1^ These authors contributed equally to this work

^*^Corresponding author. E-mail address: xc2002812@126.com

^**^Corresponding author. E-mail address: zhengyudong@mater.ustb.edu.cn

**Table S1.** Component of SPT hydrogels

| Sample Name | SA: DMTMM  (n/n) | TP (wt%) | Inorganic nanoparticles (wt%) | TPL  (μg/mL) |
| --- | --- | --- | --- | --- |
| SPT-1’ | 1:1 | 0.25 | 0 | 0 |
| SPT-2’ |  | 0.75 |  |  |
| SPT-1 / SPT1@TPL | 1:1 | 0.25 | 2  (HA: CaCO_3_=1:1) | 0 / 50 |
| SPT-2 / SPT2@TPL | 1:1 | 0.75 |  |  |
| SPT-3 / SPT3@TPL | 1:0.5 | 0.25 |  |  |
| SPT-4 / SPT4@TPL | 1:0.5 | 0.75 |  |  |

**Table S2.** Sequence of Primers for RT-qPCR

| **Genes** | **Forward primer** | **Reverse primer** |
| --- | --- | --- |
| iNOS | AACAACGTGGAGAAAACCCCA | GGGTCGATGGAGTCACATGC |
| TNF-α | GGCTCCCTCTCATCAGTTCC | CGCTTGGTGGTTTGCTACG |
| Arg-1 | CATATCTGCCAAGGACATCG | GGTCTCTTCCATCACTTTGC |
| IL-10 | GCTGTCATCGATTTCTCCCCT | AGATGTCAAACTCATTCATGGCC |


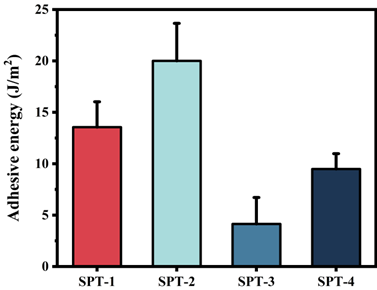


**Figure S1.** Adhesion energy of SPT hydrogels


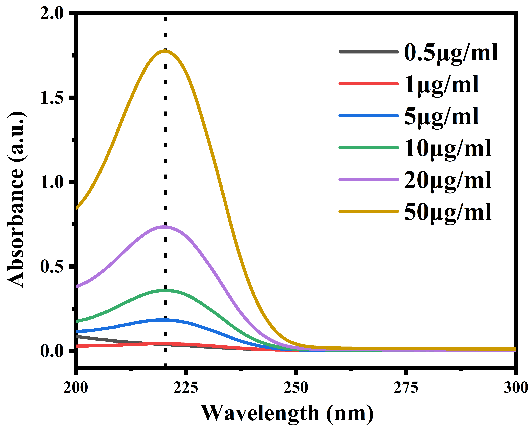

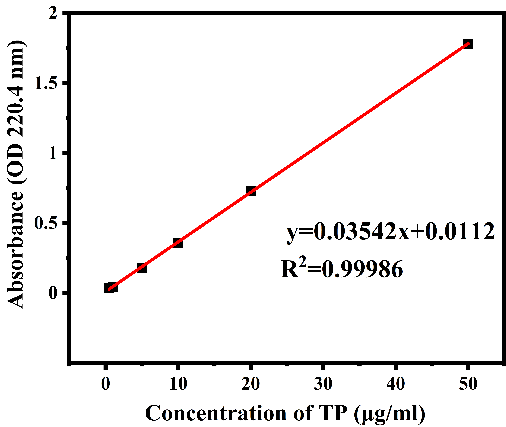


**Figure S2.** TPL standard curve measurement


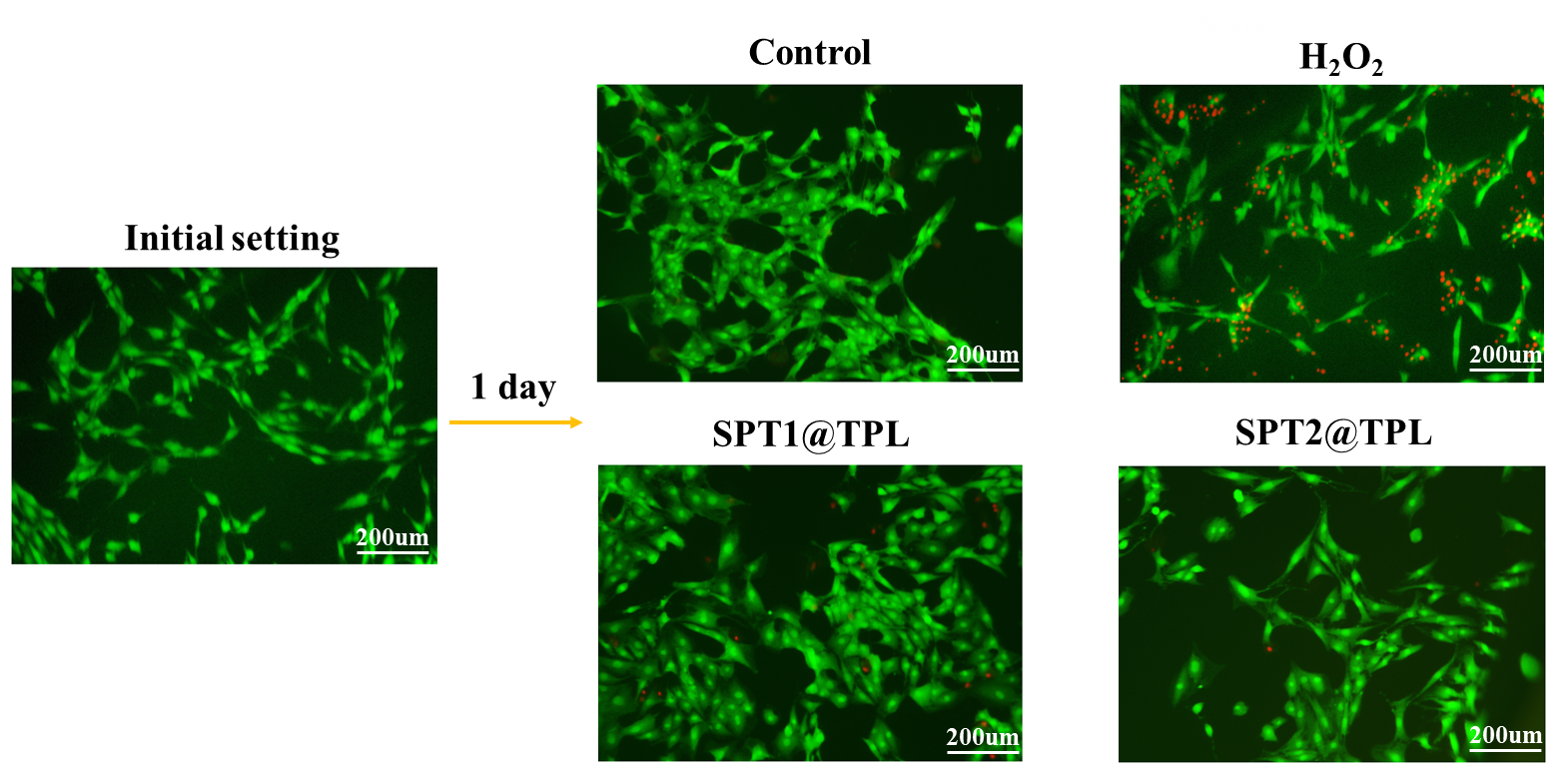


Figure S3. Live/ dead cells staining in high ROS level


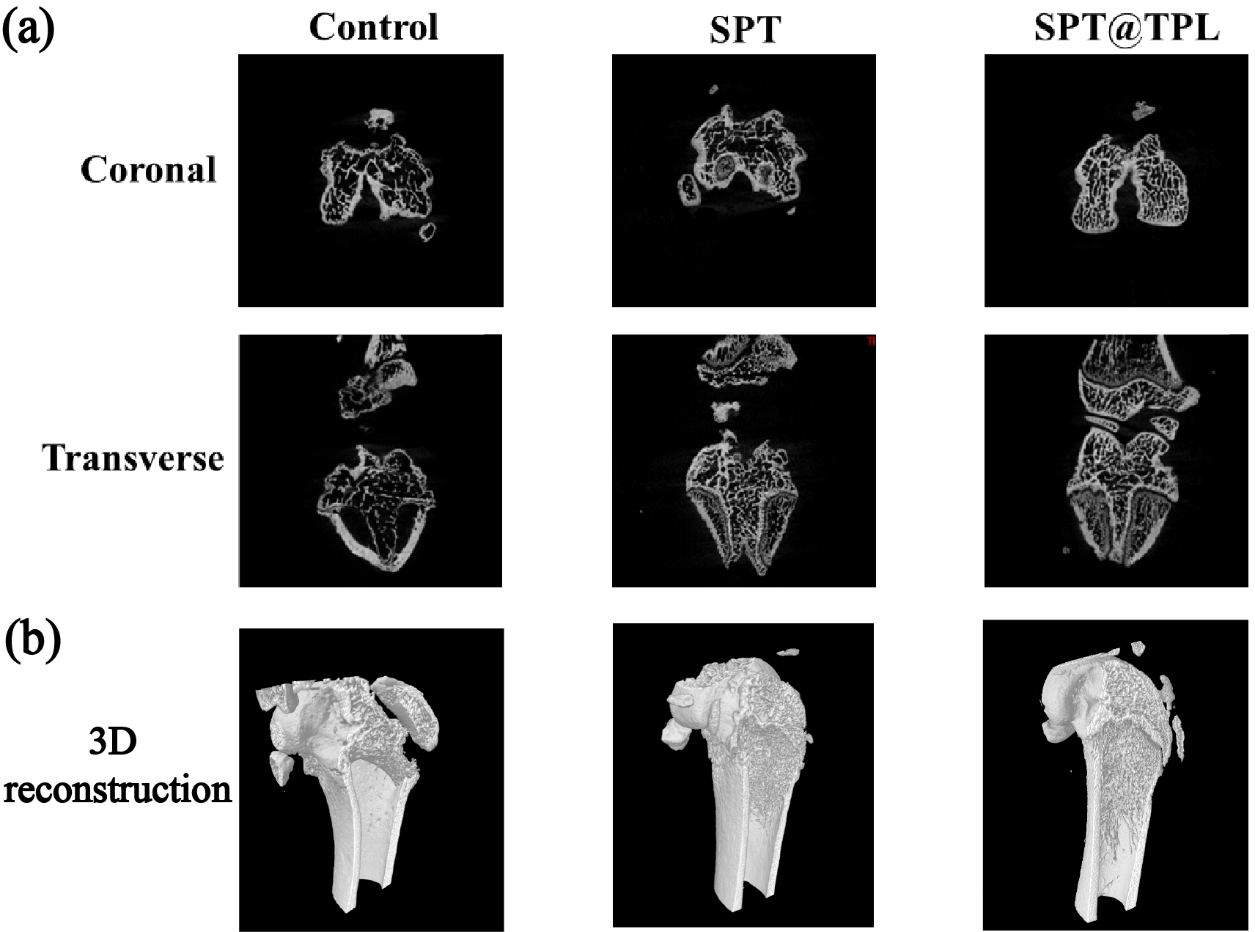


Figure S4. (a) Micro-CT images from different views and (b) Micro-CT 3D reconstruction images of femoral condyle after two months.
